# Analysis of 2,5-dimethoxy-amphetamines and 2,5-dimethoxy-phenethylamines aiming their determination in biological matrices: a review

**DOI:** 10.1007/s11419-022-00638-6

**Published:** 2022-09-14

**Authors:** Maria Nieddu, Elena Baralla, Federica Sodano, Gianpiero Boatto

**Affiliations:** 1 Department of Chemistry and Pharmacy, University of Sassari, 07100 Sassari, Italy; 2 Department of Veterinary Medicine, University of Sassari, 07100 Sassari, Italy; 3 Department of Pharmacy, University of Naples Federico II, 80131 Naples, Italy

**Keywords:** 2,5-Dimethoxy-amphetamines and -phenethylamines, Hallucinatory designer drugs, Biological matrices, LC–MS/MS, GC–MS/MS

## Abstract

**Purpose:**

The present review aims to provide an overview of methods for the quantification of 2,5-dimethoxy-amphetamines and -phenethylamines in different biological matrices, both traditional and alternative ones.

**Methods:**

A complete literature search was carried out with PubMed, Scopus and the World Wide Web using relevant keywords, e.g., designer drugs, amphetamines, phenethylamines, and biological matrices.

**Results:**

Synthetic phenethylamines represent one of the largest classes of “designer drugs”, obtained through chemical structure modifications of psychoactive substances to increase their pharmacological activities. This practice is also favored by the fact that every new synthetic compound is not considered illegal by existing legislation. Generally, in a toxicological laboratory, the first monitoring of drugs of abuse is made by rapid screening tests that sometimes can occur in false positive or false negative results. To reduce evaluation errors, it is mandatory to submit the positive samples to confirmatory methods, such as gas chromatography or liquid chromatography combined to mass spectrometry, for a more specific qualitative and quantitative analysis.

**Conclusions:**

This review highlights the great need for updated comprehensive analytical methods, particularly when analyzing biological matrices, both traditional and alternative ones, for the search of newly emerging designer drugs.

## Introduction

Phenethylamines are a class of synthetic compounds with a chemical structure similar to monoamines and with stimulant activities on the central nervous system due to the increase of monoaminergic transmission. Amphetamine represents the prototype of this class of compounds, and through its structural modifications, it is possible to obtain a significant number of novel related products, known as designer drugs, even with greater intensities of desired effects [1]. These substances have often unknown hazardous profiles and can lead to devastating health consequences for abusers. Because of their chemical structures similar to amphetamine, mainly sympathomimetic adverse effects can be expected after their consumption (e.g., anxiety, palpitations, insomnia, hyperthermia, dry mouth, hypertension, tachycardia, anorexia, nausea and abdominal pain) [2, 3]. In severe cases, amphetamine derivatives have been associated with serious adverse effects such as coma, seizures, cerebral haemorrhage, cardiac toxicity until deaths [4]. Fatal cases were related to specific compounds [5–9], while, for the majority of cases of intoxication, a positive outcome was reported [4, 10–12]. Chemical structure modifications are commonly adopted in the black market and the number of new psychoactive substances (NPS) is constantly growing. At the end of 2021, the European monitoring centre for drug and drug addiction (EMCDDA) had monitored around 880 NPS, 106 of which were phenethylamines [13], a trend very similar to those of 2020 [14]. The number of cases involving phenethylamine-derivatives significantly increased over the last decade [13, 14].

The wide distribution of these compounds is favoured by the fact that, despite having the same or greater psychotropic effects of illegal substances, they are not considered as illicit until they are officially recognized as such by the existing legislation. Generally, designer drugs are easy to produce, and the continuous increase in the number of NPS makes it difficult for clinicians and authorities to stay ahead informed. In order to limit this phenomenon, laws are continuously updating, and clinical and forensic laboratories are being equipped with increasingly reliable analytical methods to detect new substances.

According to the European drug report 2021, amphetamines are the second most consumed stimulant drugs in Europe after cocaine [13]. Since the early 1980s the term “amphetamine designer drugs” has been introduced to indicate new substances, structurally similar to amphetamine, but with enhanced psychoactive effects.

**Fig. 1:**
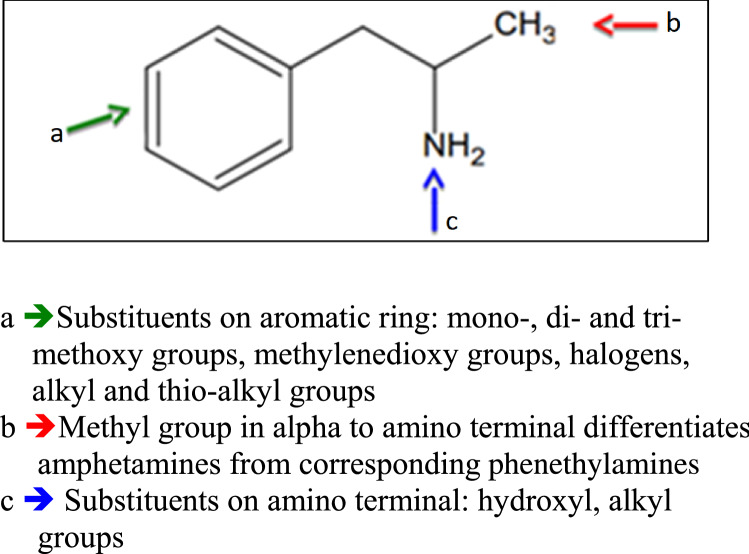
Structural modifications of amphetamine molecule

Alexander Shulgin in his two books, PIHKAL (Phenethylamines I Have Known and Loved, 1991) and TIHKAL (Tryptamines I Have Known and loved, 1997), reported the synthetic methods for over 200 new different amphetamine designer drugs [2, 15]. The chemical changes of amphetamine structure can occur in different positions, and this can affect the psychotropic activities of compounds (Fig. 1). All changes in the amphetamine basic structure led to new substances not considered illegal until they are included in the list of narcotic substances.

The presence of a methyl group in alpha position to the amino terminal moiety is typical of the amphetamines’ class, and differentiates them from the corresponding phenylethylamines.

The substitutions on the aromatic ring include mono-, di- and tri-methoxy groups in different positions. Among the methoxy-substituted derivatives, the most active compounds are those with two methoxy groups in 2,5-position (2,5-dimethoxyamphetamine, 2,5-DMA); further substitutions can occur in 4-position with halogens or alkyl groups (DOx series).

The progenitor of DOx series is the 2,5-dimethoxy-4-methylamphetamine (DOM), where the introduction of a methyl group in 4-position enhances its potency by more than one order of magnitude when compared to 2,5-DMA. The substitution of the methyl group with small alkyl groups, such as ethyl (2,5-dimethoxy-4-ethylamphetamine, DOET) and propyl (2,5-dimethoxy-4-propylamphetamine, DOPR) produces compounds with DOM like effects of equal or even greater potency than the DOM itself; further homologation to butyl (2,5-dimethoxy-4-buthylamphetamine, DOBU) decreases potency. The relative potencies of these agents, when compared to 2,5-DMA, are: 2,5-DMA < DOM < DOET < DOPR > DOBU [16].

The presence in 4-position of halogen substituents such as bromine (2,5-dimethoxy-4-bromoamphetamine, DOB), iodine (2,5-dimethoxy-4-iodoamphetamine, DOI) and chlorine (2,5-dimethoxy-4-chloroamphetamine, DOC) determines an increase in potency with respect to unsubstituted 2,5-DMA.

Even the introduction of thio-alkyl groups in the 4-position of the aromatic ring, determines an increase in potency and leads to a series of compounds denominated as “ALEPH” [2].

The analogues 2,5-dimethoxy-phenethylamines are commonly defined with the term “2C”, acronym invented by Shulgin to describe the two carbons between the amino group and the benzene ring in the chemical structure (2C-x series) [2]. The 2C derivatives thioalkyl-substituted are instead identified as “2C-T”. The first compound of the 2C series, synthesized by Shulgin in 1974, is the 2,5-dimethoxy-4-bromophenethylamine (2C-B), which appeared in the United States (US) in the second half of the 1980s. Beside the 2C-B, Shulgin described in his books the syntheses of numerous 2C compounds, many of them classified as controlled substances in the US [17] and Italy [18]: some examples are 2,5-dimethoxy-4-ethylthiophenethylamine (2C-T-2), 2,5-dimethoxy-4-(n)-propylthiophenethylamine (2C-T-7) and 2,5-dimethoxy-4-iodophenethylamine (2C-I), that, being more lipophilic than the amphetamine, show hallucinatory and stimulating effects more powerful than 2C-B [2].

Various studies on extending a lipophilic 4-substituent in 2,5-dimethoxyphenethylamines are reported [19–21]. Shulgin et al. [2] reported that the 4-substitution of 2,5-dimethoxyphenethylamines with a small lipophilic substituent induces potent psychedelic effects in humans. Many 4-substituted 2,5-dimethoxyphenethylamines potently activate the serotonin 5-HT_2_ receptors [20, 22, 23]. Kolaczynska et al. [24] confirmed that compounds containing small lipophilic substituents (halogen, methyl, CF_3_ etc.) on the 4-position exhibit agonist properties toward 5-HT_2_ receptors. Conversely, phenethylamines which contain bulky lipophilic 4-substituents exhibited serotonin 5-HT_2_ antagonist activity [19, 20].

In 2012, there was the introduction to the illicit market of *N*-benzyl-phenethylamine derivatives where the NH_2_ terminal is substituted with a 2-methoxybenzyl group (NBOMe family); the synthesis of this class of hallucinogen compounds was first reported in 2011 by scientific literature [25]. They act as powerful serotonin receptor agonists with psychedelic-hallucinogenic effects, sometimes associated with stimulant or empathogenic effects [26].

The 4-iodo-2,5-dimethoxy-*N*-(2-methoxybenzyl)phenethylamine (25I-NBOMe) is the prototype of this expanding class, and it is up to 16 times more active than the well-known 2C-I analogue amphetamine [27]. In 2014, seven NBOMe variants seized from the recreational drug market have been described [28]. Three of them, including 25I-NBOMe, the 4-chloro-2,5-dimethoxy-*N*-(2-methoxybenzyl)phenethylamine (25C-NBOMe), and the 4-bromo-2,5-dimethoxy-*N*-(2-methoxybenzyl)phenethylamine (25B-NBOMe) were scheduled both in the US and in Italy [17, 18]. Recently, several studies have focused on the analytical differentiation for new regioisomeric methoxybenzyl and dimethoxybenzyl analogues of 25-NBOMe compounds [29–31]. Most of these compounds are not currently known and diffused drugs of abuse.

Since 2015, a new class of recreational drugs, the 2,5-dimethoxy-*N*-(2-hydroxybenzyl)phenethylamines (NBOHs), structurally related to NBOMes, started appearing on the market as legal alternative to NBOMe drugs. The addition of the *N*-hydroxybenzyl moiety to the 2C core structure increases the selectivity of the NBOHs towards the 5-HT_2A_ receptors.

The first compound of the NBOH series, identified in Brazil in 2017 [32], was originally misidentified as 2C-I, because thermal conditions of gas-chromatography analysis, employed for identification, caused the degradation of the 4-iodo-2,5-dimethoxy-*N*-(2-hydroxybenzyl)phenethylamine (25I-NBOH) to the corresponding 2C compound [33]. Scientific data regarding NBOMe and NBOH compounds are constantly updating. Some metabolism studies on mice and human liver microsomes demonstrated that NBOMes readily converted into corresponding NBOH compounds [34, 35]. Yu et al. [36] proposed the creation of a MS/MS database for molecular networking as a screening method for detecting unknown emerging designer drugs. The method is based on the fact that compounds that share a structural backbone exhibit a common and characteristic MS/MS fragmentation pattern. Therefore, the organization of their fragmentation data using bioinformatics can allow assigning them the class to which they belong. The application on urine samples spiked with NBOMe derivatives showed the feasibility of this method for detecting unknown NBOMes and NBOHs in biological samples.

In the last years, other NBOH drugs (4-bromo-2,5-dimethoxy-*N*-(2-hydroxybenzyl)phenethylamine 25B-NBOH, 4-chloro-2,5-dimethoxy-*N*-(2-hydroxybenzyl)phenethylamine 25C-NBOH, 4-ethyl-2,5-dimethoxy-*N*-(2-hydroxybenzyl)phenethylamine 25E-NBOH and 2,5-dimethoxy-*N*-(2-hydroxybenzyl)phenethylamine 25H-NBOH) were identified in blotter papers in Brazil [37] and Singapore [38].

Following these findings, some NBOHs have been inserted in the list of prohibited substances in Brazil (25I-NBOH, 25B-NBOH, 25C-NBOH, 25E-NBOH and 25H-NBOH) [39] and in Italy (25B-NBOH and 25E-NBOH) [18], while, they are still legal in the US.

The majority of forensic toxicology laboratories commonly employ a preliminary screening test in order to detect drugs of abuse in biological matrices. As the preliminary immunoassays cannot differentiate between all amphetamine derivatives, it is necessary to use confirmatory methods even for the screening tests. The validation of analytical methods for determination of phenethylamines has exponentially increased over the years, alongside with the rapid growth in the number of clinical and forensic positive cases. Therefore, the presence of updated efficient procedures for their identification in biological matrices is an essential goal for toxicological analysis.

The present review aims to provide an overview of the analytical methods to confirm the presence of 2,5-dimethoxy-amphetamines and -phenethylamines in biological specimens. The substances investigated in this review and related analytical methods are reported in Table 1.

**Table 1:** List of investigated 2,5-dimethoxy-amphetamines and -phenethylamines, and related analytical methods

Abbreviated name	Formal name	Scheduled in US[17]	Scheduled in Italy [18]	Reference	Analytical method	Biological matrix	LOQ
DMA	2,5-Dimethoxyamphetamine	X	X	[109]	GC–MS	Plasma, urine, hair	n.d
				[111]	CE–DAD	Blood	4300 ng/mL
				[112]	CE–MS	Urine	4.0 ng/mL
				[12]	LC–MS/MS	Plasma, urine	10.0 ng/mL
				[88]	LC–MS/MS	Hair	0.18 ng/mg
				[134]	LC–MS/MS	Hair	0.01 ng/mg^a^
				[139]	LC–MS/MS	Amniotic fluid	20.0 ng/mL
				[140]	LC–MS/MS	Oral fluid	9.5 ng/mL
				[151]	LC–MS/MS	Urine	1.0 ng/mL
				[153]	LC–MS/MS	Blood, urine	4.0 ng/mL
DOB	2,5-Dimethoxy-4-bromoamphetamine	X	X	[109]	GC–MS	Plasma, urine, hair	n.d
				[111]	CE–DAD	Blood	4500 ng/mL
				[112]	CE–MS	Urine	9.0 ng/mL
				[12]	LC–MS/MS	Plasma, urine	10.0 ng/mL
				[132]	LC–MS/MS	Hair	0.05 ng/mg
				[88]	LC–MS/MS	Hair	0.13 ng/mg
				[139]	LC–MS/MS	Amniotic fluid	19.0 ng/mL
				[140]	LC–MS/MS	Oral fluid	8.5 ng/mL
				[144]	LC–MS/MS	Blood, urine	2.5 ng/mL^a^
				[145]	LC–MS/MS	Blood	15.0 ng/mL^a^
				[151]	LC–MS/MS	Urine	1.0 ng/mL
DOC	2,5-Dimethoxy-4-chloroamphetamine		X	[111]	CE–DAD	Blood	4800 ng/mL
				[112]	CE–MS	Urine	4.4 ng/mL
				[12]	LC–MS/MS	Plasma, urine	10.0 ng/mL
				[145]	LC–MS/MS	Blood	n.d
				[151]	LC–MS/MS	Urine	1.0 ng/mL
DOET	2,5-Dimethoxy-4-ethylamphetamine	X	X	[123]	CE–MS	Urine	15.3 ng/mL
				[88]	LC–MS/MS	Hair	0.12 ng/mg
				[12]	LC–MS/MS	Plasma, urine	10.0 ng/mL
				[139]	LC–MS/MS	Amniotic fluid	15.0 ng/mL
				[140]	LC–MS/MS	Oral fluid	6.3 ng/mL
				[151]	LC–MS/MS	Urine	1.0 ng/mL
DOI	2,5-Dimethoxy-4-iodoamphetamine			[111]	CE–DAD	Blood	2100 ng/mL
				[112]	CE–MS	Urine	6.5 ng/mL
				[12]	LC–MS/MS	Plasma, urine	10.0 ng/mL
				[144]	LC–MS/MS	Blood, urine	1.0 ng/mL
				[145]	LC–MS/MS	Blood	15.0 ng/mL^a^
				[151]	LC–MS/MS	Urine	2.5 ng/mL^a^
DOM	2,5-Dimethoxy-4-methylamphetamine	X	X	[109]	GC–MS	Plasma, urine, hair	n.d
				[111]	CE–DAD	Blood	6400 ng/mL
				[112]	CE–MS	Urine	12.9 ng/mL
				[123]	CE–MS	Urine	13.8 ng/mL
				[12]	LC–MS/MS	Plasma, urine	10.0 ng/mL
				[132]	LC–MS/MS	Hair	0.05 ng/mg
				[88]	LC–MS/MS	Hair	0.09 ng/mg
				[139]	LC–MS/MS	Amniotic fluid	19.0 ng/mL
				[140]	LC–MS/MS	Oral fluid	6.4 ng/mL
				[151]	LC–MS/MS	Urine	1.0 ng/mL
DON	2,5-Dimethoxy-4-nitroamphetamine			[111]	CE–DAD	Blood	4900 ng/mL
				[112]	CE–MS	Urine	14.0 ng/mL
DOPR	2,5-Dimethoxy-4-propylamphetamine			[123]	CE–MS	Urine	14.l ng/mL
2C-H	2,5-Dimethoxyphenethylamine	X	X	[130]	LC–MS/MS	Blood	0.05 ng/mL^a^
				[136]	LC–MS/MS	Hair	0.025 ng/mg
				[153]	LC–MS/MS	Blood, urine	4.0 ng/mL
2C-G	2,5-Dimethoxy-3, 4-dimethylphenethylamine			[134]	LC–MS/MS	Hair	0.01 ng/mg^a^
				[149]	LC–MS/MS	Oral fluid	1.0 ng/mL^a^
2C-D, 2C-M	2,5-Dimethoxy-4-methylphenethylamine	X	X	[108]	GC–MS	Urine	10.0 ng/mL
				[111]	CE–DAD	Blood	2500 ng/mL
				[112]	CE–MS	Urine	1.0 ng/mL
				[130]	LC–MS/MS	Blood	n.d
				[131]	LC–MS/MS	Blood, plasma, urine	10.0 ng/mL^a^
				[149]	LC–MS/MS	Oral fluid	0.1 ng/mL^a^
				[151]	LC–MS/MS	Urine	1.0 ng/mL
2C-E	2,5-Dimethoxy-4-ethylphenethylamine	X	X	[108]	GC–MS	Urine	10.0 ng/mL
				[130]	LC–MS/MS	Blood	0.84 ng/mL^a^
				[131]	LC–MS/MS	Blood, plasma, serum	25.0 ng/mL^a^
				[131]	LC–MS/MS	Urine	10.0 ng/mL^a^
				[134]	LC–MS/MS	Hair	0.01 ng/mg^a^
				[144]	LC–MS/MS	Blood, urine	2.5 ng/mL^a^
				[145]	LC–MS/MS	Blood	n.d
				[149]	LC–MS/MS	Oral fluid	1.0 ng/mL^a^
				[151]	LC–MS/MS	Urine	1.0 ng/mL
2C-P	2,5-Dimethoxy-4-propylphenethylamine			[108]	GC–MS	Urine	10.0 ng/mL
				[131]	LC–MS/MS	Blood, plasma, serum	25.0 ng/mL^a^
				[131]	LC–MS/MS	Urine	10.0 ng/mL^a^
				[133]	LC–MS/MS	Hair	0.002 ng/mg
				[155]	LC–QTOF-MS	Plasma	2.0 ng/mL^a^
2C-B	2,5-Dimethoxy-4-bromophenethylamine	X	X	[107]	GC–MS	Blood, urine	50.0 ng/mL
				[108]	GC–MS	Urine	10.0 ng/mL
				[111]	CE–DAD	Blood	2300 ng/mL
				[112]	CE–MS	Urine	5.3 ng/mL
				[130]	LC–MS/MS	Blood	0.20 ng/mL^a^
				[131]	LC–MS/MS	Blood, plasma, serum	50.0 ng/mL^a^
				[131]	LC–MS/MS	Urine	30.0 ng/mL^a^
				[88]	LC–MS/MS	Hair	0.2 ng/mg
				[133]	LC–MS/MS	Hair	0.012 ng/mg
				[134]	LC–MS/MS	Hair	0.05 ng/mg^a^
				[136]	LC–MS/MS	Hair	0.05 ng/mg
				[139]	LC–MS/MS	Amniotic fluid	19.0 ng/mL
				[140]	LC–MS/MS	Oral fluid	8.0 ng/mL
				[145]	LC–MS/MS	Blood	15.0 ng/mL^a^
				[149]	LC–MS/MS	Oral fluid	1.0 ng/mL^a^
				[151]	LC–MS/MS	Urine	1.0 ng/mL
				[152]	LC–MS/MS	Blood	10.0 ng/mL
				[153]	LC–MS/MS	Blood, urine	4.0 ng/mL
2C-C	2,5-Dimethoxy-4-chlorophenethylamine	X		[130]	LC–MS/MS	Blood	n.d
				[144]	LC–MS/MS	Blood, urine	2.5 ng/mL^a^
				[149]	LC–MS/MS	Oral fluid	1.0 ng/mL^a^
				[151]	LC–MS/MS	Urine	1.0 ng/mL
				[155]	LC–QTOF-MS	Plasma	11.0 ng/mL^a^
2C-I	2,5-Dimethoxy-4-iodophenethylamine	X	X	[108]	GC–MS	Urine	10.0 ng/mL
				[111]	CE–DAD	Blood	5900 ng/mL
				[112]	CE–MS	Urine	12.0 ng/mL
				[130]	LC–MS/MS	Blood	n.d
				[131]	LC–MS/MS	Blood, plasma, serum	25.0 ng/mL^a^
				[131]	LC–MS/MS	Urine	10.0 ng/mL^a^
				[88]	LC–MS/MS	Hair	0.19 ng/mg
				[134]	LC–MS/MS	Hair	0.01 ng/mg^a^
				[139]	LC–MS/MS	Amniotic fluid	12.0 ng/mL
				[140]	LC–MS/MS	Oral fluid	8.8 ng/mL
				[144]	LC–MS/MS	Blood, urine	2.5 ng/mL^a^
				[149]	LC–MS/MS	Oral fluid	0.1 ng/mL^a^
				[151]	LC–MS/MS	Urine	1.0 ng/mL
				[152]	LC–MS/MS	Blood	10.0 ng/mL
				[153]	LC–MS/MS	Blood, urine	4.0 ng/mL
2C-N	2,5-Dimethoxy-4-nitrophenethylamine	X		[111]	CE–DAD	Blood	7200 ng/mL
				[112]	CE–MS	Urine	9.8 ng/mL
				[130]	LC–MS/MS	Blood	n.d
				[134]	LC–MS/MS	Hair	0.05 ng/mg^a^
				[152]	LC–MS/MS	Blood	10.0 ng/mL
2C-CN	2,5-Dimethoxy-4-cyanophenethylamine			[150]	LC–MS/MS	Plasma	1.0 ng/mL
				[150]	LC–MS/MS	Brain	0.05 ng/mg
2C-T	2,5-Dimethoxy-4-methylthiophenethylamine			[117]	CE–MS	Plasma	35.6 ng/mL
				[125]	LC–MS/MS	Urine	3.6 ng/mL
				[131]	LC–MS/MS	Blood,plasma,serum	50.0 ng/mL^a^
				[131]	LC–MS/MS	Urine	30.0 ng/mL^a^
				[149]	LC–MS/MS	Oral fluid	1.0 ng/mL^a^
2CT-2	2,5-Dimethoxy-4-ethylthiophenethylamine	X	X	[108]	GC–MS	Urine	/
				[117]	CE–MS	Plasma	27.3 ng/mL
				[125]	LC–MS/MS	Urine	9.6 ng/mL
				[130]	LC–MS/MS	Blood	n.d
				[88]	LC–MS/MS	Hair	0.19 ng/mg
				[139]	LC–MS/MS	Amniotic fluid	13.0 ng/mL
				[140]	LC–MS/MS	Oral fluid	9.2 ng/mL
				[149]	LC–MS/MS	Oral fluid	1.0 ng/mL^a^
				[151]	LC–MS/MS	Urine	1.0 ng/mL
2CT-4	2,5-Dimethoxy-4-isopropylthiophenethylamine	X		[119]	CE–MS	Urine	9.1 ng/mL
				[135]	LC–MS/MS	Hair	0.05 ng/mg
				[136]	LC–MS/MS	Hair	0.02 ng/mg
				[149]	LC–MS/MS	Oral fluid	0.1 ng/mL^a^
2CT-5	2,5-Dimethoxy-4-cyclohexylthiophenethylamine			[117]	CE–MS	Plasma	43.0 ng/mL
				[125]	LC–MS/MS	Urine	4.9 ng/mL
2CT-7	2,5-Dimethoxy-4-*n*-propylthiophenethylamine	X	X	[107]	GC–MS	Blood, urine	50.0 ng/mL
				[108]	GC–MS	Urine	/
				[117]	CE–MS	Plasma	37.9 ng/mL
				[125]	LC–MS/MS	Urine	9.5 ng/mL
				[130]	LC–MS/MS	Blood	n.d
				[88]	LC–MS/MS	Hair	0.19 ng/mg
				[136]	LC–MS/MS	Hair	0.02 ng/mg
				[139]	LC–MS/MS	Amniotic fluid	14.0 ng/mL
				[140]	LC–MS/MS	Oral fluid	8.3 ng/mL
				[151]	LC–MS/MS	Urine	1.0 ng/mL
2CT-8	2,5-Dimethoxy-4-cyclopropylmethylthiophenethylamine			[119]	CE–MS	Urine	7.5 ng/mL
2CT-13	2,5-Dimethoxy-4- (2-methoxyethyl)thiophenethylamine			[119]	CE–MS	Urine	10.0 ng/mL
2CT-17	2,5-Dimethoxy-4-i-butylthiophenethylamine			[119]	CE–MS	Urine	8.9 ng/mL
ALEPH	2,5-Dimethoxy-4-methylthioamphetamine			[118]	CE–MS	Plasma	70.0 ng/mL
				[125]	LC–MS/MS	Urine	9.3 ng/mL
ALEPH-2	2,5-Dimethoxy-4-ethylthioamphetamine			[118]	CE–MS	Plasma	85.0 ng/mL
				[125]	LC–MS/MS	Urine	3.2 ng/mL
ALEPH-4	2,5-Dimethoxy-4-isopropylthioamphetamine			[120]	CE–DAD	Urine	36,000 ng/mL
ALEPH-5	2,5-Dimethoxy-4-cyclohexylthioamphetamine			[118]	CE–MS	Plasma	90.0 ng/mL
				[125]	LC–MS/MS	Urine	8.6 ng/mL
ALEPH-7	2,5-Dimethoxy-4-*n*-propylthioamphetamine			[118]	CE–MS	Plasma	59.0 ng/mL
				[125]	LC–MS/MS	Urine	7.9 ng/mL
ALEPH-8	2,5-Dimethoxy-4-cyclopropylmethylthioamphetamine			[120]	CE–DAD	Urine	65,900 ng/mL
ALEPH-13	2,5-Dimethoxy-4- (2-methoxyethyl) thioamphetamine			[120]	CE–DAD	Urine	33,400 ng/mL
ALEPH-17	2,5-Dimethoxy-4-i-butylthioamphetamine			[120]	CE–DAD	Urine	43,300 ng/mL
25H-NBOMe	2,5-Dimethoxy-*N*-(2-methoxybenzyl)phenethylamine		X	[28]	LC–MS/MS	Urine	1.0 ng/mL
				[130]	LC–MS/MS	Blood	0.13 ng/mL^a^
				[133]	LC–MS/MS	Hair	0.002 ng/mg
				[146]	LC–MS/MS	Urine	1.0 ng/mL^a^
				[149]	LC–MS/MS	Oral fluid	0.05 ng/mL^a^
				[151]	LC–MS/MS	Urine	1.0 ng/mL
25B-NBOMe	4-Bromo-2,5-dimethoxy-*N*-(2-methoxybenzyl)phenethylamine	X	X	[28]	LC–MS/MS	Urine	1.0 ng/mL
				[127]	LC–MS/MS	Serum, urine	0.025 ng/mL
				[130]	LC–MS/MS	Blood	0.21 ng/mL^a^
				[133]	LC–MS/MS	Hair	0.0082 ng/mg
				[144]	LC–MS/MS	Blood, urine	2.5 ng/mL^a^
				[145]	LC–MS/MS	Blood	0.8 ng/mL^a^
				[146]	LC–MS/MS	Urine	0.5 ng/mL^a^
				[149]	LC–MS/MS	Oral fluid	0.1 ng/mL^a^
				[151]	LC–MS/MS	Urine	1.0 ng/mL
				[152]	LC–MS/MS	Blood	1.0 ng/mL
				[153]	LC–MS/MS	Blood, urine	0.4 ng/mL
25C-NBOMe	4-Chloro-2,5-dimethoxy-*N*-(2-methoxybenzyl)phenethylamine	X	X	[129]	LC–MS/MS	Serum	0.03 ng/mL
				[28]	LC–MS/MS	Urine	1.0 ng/mL
				[130]	LC–MS/MS	Blood	n.d
				[133]	LC–MS/MS	Hair	0.003 ng/mg
				[134]	LC–MS/MS	Hair	0.001 ng/mg^a^
				[144]	LC–MS/MS	Blood, urine	2.5 ng/mL^a^
				[145]	LC–MS/MS	Blood	0.7 ng/mL^a^
				[146]	LC–MS/MS	Urine	1.0 ng/mL^a^
				[149]	LC–MS/MS	Oral fluid	1.0 ng/mL^a^
				[151]	LC–MS/MS	Urine	0.05 ng/mL
				[152]	LC–MS/MS	Blood	1.0 ng/mL
				[153]	LC–MS/MS	Blood, urine	0.4 ng/mL
				[155]	LC–QTOF-MS	Plasma	26.0 ng/mL^a^
25D-NBOMe	4-Methyl-2,5-dimethoxy-*N*-(2-methoxybenzyl)phenethylamine			[28]	LC–MS/MS	Urine	1.0 ng/mL
				[149]	LC–MS/MS	Oral fluid	0.1 ng/mL^a^
				[151]	LC–MS/MS	Urine	1.0 ng/mL
				[155]	LC–QTOF-MS	Plasma	17.0 ng/mL^a^
25E-NBOMe	4-Ethyl-2,5-dimethoxy-*N*-(2-methoxybenzyl)phenethylamine			[149]	LC–MS/MS	Oral fluid	1.0 ng/mL^a^
				[153]	LC–MS/MS	Blood, urine	0.4 ng/mL
25G-NBOMe	3,4-Dimethyl-2,5-dimethoxy-*N*-(2-methoxybenzyl)phenethylamine			[28]	LC–MS/MS	Urine	1.0 ng/mL
				[149]	LC–MS/MS	Oral fluid	0.1 ng/mL^a^
				[151]	LC–MS/MS	Urine	1.0 ng/mL
25I-NBOMe	4-Iodo-2,5-dimethoxy-*N*-(2-methoxybenzyl)phenethylamine	X	X	[129]	LC–MS/MS	Serum	0.03 ng/mL
				[28]	LC–MS/MS	Urine	1.0 ng/mL
				[9]	LC–MS/MS	Blood, urine, bile	0.025 ng/mL
				[130]	LC–MS/MS	Blood	0.09 ng/mL^a^
				[133]	LC–MS/MS	Hair	0.003 ng/mg
				[134]	LC–MS/MS	Hair	0.001 ng/mg^a^
				[136]	LC–MS/MS	Hair	0.05 ng/mg
				[144]	LC–MS/MS	Blood, urine	2.5 ng/mL^a^
				[145]	LC–MS/MS	Blood	0.5 ng/mL^a^
				[146]	LC–MS/MS	Urine	0.5 ng/mL^a^
				[149]	LC–MS/MS	Oral fluid	0.05 ng/mL^a^
				[151]	LC–MS/MS	Urine	1.0 ng/mL
				[152]	LC–MS/MS	Blood	1.0 ng/mL
				[153]	LC–MS/MS	Blood, urine	0.4 ng/mL
				[155]	LC–QTOF-MS	Plasma	27.0 ng/mL^a^
25N-NBOMe	4-Nitro-2,5-dimethoxy-*N*-(2-methoxybenzyl) phenethylamine			[149]	LC–MS/MS	Oral fluid	0.05 ng/mL^a^
				[151]	LC–MS/MS	Urine	1.0 ng/mL
25P-NBOMe	4-Propyl-2,5-dimethoxy-*N*-(2-methoxybenzyl) phenethylamine			[151]	LC–MS/MS	Urine	1.0 ng/mL
25T-NBOMe	4-Methylthio-2,5-dimethoxy-*N*-(2-methoxybenzyl) phenethylamine			[28]	LC–MS/MS	Urine	1.0 ng/mL
25T2-NBOMe	(4-Ethylthio)-2,5-dimethoxy-*N*-(2-methoxybenzyl)phenethylamine			[28]	LC–MS/MS	Urine	1.0 ng/mL
				[149]	LC–MS/MS	Oral fluid	0.05 ng/mL^a^
				[151]	LC–MS/MS	Urine	1.0 ng/mL
25T4-NBOMe	4-Isopropylthio-2,5-dimethoxy-*N*-(2-methoxybenzyl)phenethylamine			[28]	LC–MS/MS	Urine	1.0 ng/mL
				[151]	LC–MS/MS	Urine	1.0 ng/mL
25T7-NBOMe	4-Propylthio-2,5-dimethoxy-*N*-(2-methoxybenzyl)phenethylamine			[28]	LC–MS/MS	Urine	1.0 ng/mL
				[151]	LC–MS/MS	Urine	1.0 ng/mL
25H-NBOH	2,5-Dimethoxy-*N*-(2-hydroxybenzyl)phenethylamine			[146]	LC–MS/MS	Urine	1.0 ng/mL^a^
25B-NBOH	4-Bromo-2,5-dimethoxy-*N*-(2-hydroxybenzyl)phenethylamine		X	[145]	LC–MS/MS	Blood	n.d
				[146]	LC–MS/MS	Urine	0.5 ng/mL^a^
				[149]	LC–MS/MS	Oral fluid	0.1 ng/mL^a^
				[153]	LC–MS/MS	Blood, urine	0.4 ng/mL
25C-NBOH	4-Chloro-2,5-dimethoxy-*N*-(2-hydroxybenzyl)phenethylamine			[145]	LC–MS/MS	Blood	n.d
				[146]	LC–MS/MS	Urine	0.5 ng/mL^a^
				[149]	LC–MS/MS	Oral fluid	0.1 ng/mL^a^
				[153]	LC–MS/MS	Blood, urine	0.4 ng/mL
25E-NBOH	4-Ethyl-2,5-dimethoxy-*N*-(2-hydroxybenzyl)phenethylamine		X	[145]	LC–MS/MS	Blood	n.d
				[149]	LC–MS/MS	Oral fluid	0.05 ng/mL^a^
				[153]	LC–MS/MS	Blood, urine	0.4 ng/mL
25I-NBOH	4-Iodo-2,5-dimethoxy-*N*-(2-hydroxybenzyl)phenethylamine			[144]	LC–MS/MS	Blood, urine	2.5 ng/mL^a^
				[145]	LC–MS/MS	Blood	n.d
				[146]	LC–MS/MS	Urine	0.5 ng/mL^a^
				[149]	LC–MS/MS	Oral fluid	0.05 ng/mL^a^
				[153]	LC–MS/MS	Blood, urine	0.4 ng/mL
25CN-NBOH	4-Cyano-2,5-dimethoxy-*N*-(2-hydroxybenzyl)phenethylamine			[150]	LC–MS/MS	Plasma	1.0 ng/mL
				[150]	LC–MS/MS	Brain	0.05 ng/mg

*GC–MS *gas chromatography–mass spectrometry, *CE–DAD* capillary electrophoresis–diode array detection, *CE–MS* capillary electrophoresis–mass spectrometry,

*LC–MS/MS *liquid chromatography–tandem mass spectrometry, *LC–QTOF-MS *liquid chromatography–quadrupole time-of-flight mass spectrometry, LOQ limit of quantification

*n.d.* not determined

^a^Limit of detection in place of LOQ

## Biological specimens used for the analysis of drugs of abuse

The choice of the biological matrix depends on the purpose of the survey and on the basis of information to achieve.

Blood and urine are historically the most widely used biological matrices for toxicological analysis. Blood or plasma analysis is preferred in the case of acute intoxication, while in chronic intoxications it is important to evaluate both the presence of the target analyte and its metabolites in urine. Moreover, in the last years, researchers have started to use alternative biological matrices, as hair or oral fluid, that allow easier and less invasive samplings. Each of these matrices shows advantages and disadvantages, and several studies are actually carried out, confirming its usefulness in support of traditional biological specimens [40–42].

### Urine

Urine is the matrix of choice for preliminary screening methods because it permits to obtain a considerable volume of sample with a noninvasive collection procedure, particularly useful in the cases where the repetition of the analysis is necessary [40]. Furthermore, urine analysis allows the qualitative detection of a wide range of substances and their metabolites even several days after intake, depending on their half-life [43]. The presence of a drug in the urine varies according to the dose taken, the frequency and mode of intake and the time elapsed between consumption and sampling.

The analysis of substances of abuse in urine ranges from the toxicological control of subjects undergoing drugs detoxification to investigation in case of fatal intoxications. Urine analysis is even used in monitoring programs of subjects suspected of working under the influence of drugs.

The main problem associated with urine sampling regards the possibility of alteration and/or replacement of the sample. To avoid this, it is necessary to have adequate sampling rooms and qualified medical personnel. The most common method of sample manumission is the dilution, "In vitro" by adding diluents, or "In vivo" by excessive intake of diuretics, water or other liquids [44]. The final aim is to reduce the drug concentration and produce a negative response [45]. Another way consists in replacing the original sample with one drug-free or in adding adulterant substances that can interfere with the analysis by reducing the drug concentration. For example, oxidant agents can destroy the target molecule, making it undetectable by analytical methods [46]. A great number of products, specifically formulated for urine adulteration, are readily available on the Internet [47–51].

Urine sample integrity testing procedure has been published in the US Federal Registry and approved by the US Substance and Mental Health Services Administration (SAMSHA) [52, 53]. Another disadvantage of urine analysis is that the presence of drug in urine does not necessarily indicate an use immediately preceding, or even at few hours before using.

### Blood

Blood analysis, unlike urine, permits to establish or exclude the recent intake of a substance and it is directly related to the psychophysical state of the subject at the time of collection. For this reason, it represents the biological matrix of choice to evaluate a short-term intake. For example, blood analysis is widely used to investigate the driving under the influence of drugs in case of road accidents; it is also useful for assessing the intake of psychotropic substances in workplaces or in fatal intoxications.

An additional advantage is that blood is not an alterable matrix, and this is the main reason why it is considered the first choice in forensic investigations. Unfortunately, every substance is detectable only within a short period of time, depending on its plasma half-life; the drug concentration can significantly decrease within a few hours. The knowledge of the pharmacokinetics of a specific substance allows evaluating a recent intake.

Studies on designer drugs of amphetamines have shown that the maximum plasma concentration is reached in 2–4 h, while the plasma half-life is approximately 5–10 h [54]. The main disadvantage of blood sampling is that it is an invasive procedure and must be performed by trained personnel. Therefore, it is not suitable to on-site sampling.

### Oral fluid

In the last decades, toxicological analysis has been directed towards unconventional biological matrices such as oral fluid and hair. The use of oral fluid was officially approved in 2011 by SAMHSA [55]. Its main application concerns with detection of drugs of abuse such as amphetamines, ketamine, cocaine, opiates, cannabis and benzodiazepines [56–62].

The oral fluid analysis allows to identify the recent intake of a substance, even after few hours from ingestion, depending on its bioavailability [63, 64]. For this reason, it is increasingly used, alternatively to blood and urine, in order to detect the consumption of drugs in workplaces, in clinical toxicological analysis, in criminal fields and in monitoring programs of driving under the influence of drugs (DUID program) [65–68].

Indeed, the oral fluid concentration reflects the drug concentration in blood being better than urine, and, therefore, makes it possible to detect a relatively recent intake [69, 70]. Quantitative measurements in oral fluid can therefore be a valuable tool for determining the degree of exposure to a substance at the time of sampling [41].

For most substances, the concentration in this matrix can be estimated on the basis of oral fluid pH in respect to plasma pH, and the pKa of the drug [71, 72]. Generally, basic drugs reach higher concentrations in oral fluid than in plasma [73, 74]. Most psychotropic substances, e.g., opiates, amphetamines and cocaine, are basic, and thus, in oral fluid, they can present concentrations higher than those found in blood [69, 75–78].

The advantages of this matrix are the great simplicity and noninvasiveness of sampling, which can be carried out anywhere and without the supervision of specialized medical personnel; however, it is preferable to carry out the sample collection under supervision of trained personnel, to prevent adulteration or replacement of the sample itself [41]. The oral fluid can be collected through the suitable devices equipped with a swab for the sample collection. The procedure and devices used can significantly affect the concentration and recovery of different substances [79–81].

Compared to blood and urine, oral fluid has the disadvantage of providing a smaller sample volume. Sometimes, it can be difficult to collect a sufficient volume of sample, due to mouth dryness, a phenomenon relatively common that can be caused by different physiological or external factors, as the anxiety of the collection procedure, the poor hydration of the subject, but also the intake of drugs or substances that can restrict salivary flow [57]. On the other hand, some drugs, such as clonidine, pilocarpine and beta-2 stimulants, increase salivary flow, causing a dilution of drugs concentration contained in it. Smoking or food intake can also contaminate the oral fluid, causing an altered response [82]. Rinsing the mouth before sample collection can help to reduce any residual contamination [40].

### Hair

Hair is an interesting specimen for measuring chronic exposure and has been demonstrated to be a powerful tool, in forensic and clinical toxicology, for detecting therapeutic and illicit drug use, in defining the history of drug abuse and in postmortem toxicology [83–85].

It is a complex matrix in which, drugs and exogenous compounds may be incorporated, depending on their chemical nature [86]. Despite this complexity, hair has numerous advantages over traditional matrices as blood or urine [42, 87]. First, it allows the investigation of a longer detection window (months to years), as the substances remain in hair for a long time without significant loss/degradation [88]. Furthermore, hair analysis has been used to demonstrate a chronic drug use as it is less affected by adulteration or short-term abstinence than other matrices. Moreover, its sampling is noninvasive, as collecting head hair is less intrusive and causes less embarrassment. Additionally, the sample is durable, stable and easy to store [88].

Researchers have demonstrated that basic drugs are incorporated into hair to a greater extent than neutral or acidic drugs [42]. Hair pigmentation (melanin concentration) affects the extent and rate of drug incorporation into hair [89, 90]. Basic drugs have been shown to have higher concentrations in pigmented hair (higher melanin concentrations) as compared with non-pigmented hair [91–93]. In contrast, hair concentrations of acidic or neutral drugs have not been correlated with melanin concentrations [92]. Drugs with cationic properties, such as phenethylamines, appear to bind melanin through the establishment of electrostatic forces between the negative charges of the matrix and the positive charges on the molecule [94]. This suggests that melanin concentration is an important factor when determining drug concentrations in hair at physiological pH.

### Amniotic fluid

Foetal exposure to drugs of abuse can be diagnosed through maternal history and drugs detection in either maternal and/or neonatal matrices. In addition to traditional specimens such as maternal blood and urine, there are many others available matrices, such as meconium, umbilical cord and amniotic fluid. Amniotic fluid is essentially a filtrate of maternal blood, and it acts as a foetal excretion reservoir, accumulating drugs through gestation. The drugs diffuse in it across the placenta and can reach the foetus through two routes: oral ingestion of the amniotic fluid and transdermal diffusion; the latter is particularly relevant in the early stage of pregnancy, because the skin is less developed. The great disadvantage of this matrix is that its collection is invasive. However, an aliquot of sample can be collected during amniocentesis, a medical procedure performed to detect genetic disorders [95]. Although the sample collection is more troublesome than other conventional matrices, this matrix is unique because it can measure second/third trimester pregnancy drug exposure. Furthermore, it gives a more direct measure of foetal exposure than maternal blood as it indicates real drugs concentration that has passed the placental barrier. Phenethylamines can readily pass through the placental barrier, reaching the foetal compartment and causing harmful side effects [96].

## Analytical methods for simultaneous determination of 2,5-dimethoxy-amphetamines and -phenethylamines in biological matrices

### Gas chromatography

Gas chromatography–mass spectrometry (GC–MS) analysis has been ever since the most largely used for investigations concerning metabolism. In literature there are several studies about detection of 2,5-dimethoxy-amphetamines and -phenethylamines and their metabolites in urine, but they did not report method validation [97–106]. Much less publications dealt with simultaneous screening and/or validated quantification of these designer drugs in biological matrices by GC–MS analysis.

A screening GC–MS method was developed by Vorce and Sklerov [107] for measuring some tryptamines and phenethylamines (including 2C-B and 2C-T-7) in blood and urine, using derivatization with pentafluoropropionic anhydride (PFPA) to enhance the sensitivity. The method demonstrated linearity between 50 and 1000 ng/mL and it was successfully applied to real blood and urine samples.

Habdrova et al. [108] described a method for quantification of several designer drugs of the 2C series (2C-D, 2C-E, 2C-P, 2C-B, 2C-I, 2C-T-2, 2C-T-7) in human blood/plasma by GC–MS. The GC analysis required a derivatization with heptafluorobutyric anhydride. Biological fluids were extracted by mixed-mode solid-phase extraction. The method was fully validated according to international guidelines except for 2C-T-2 and 2C-T-7; validation data for these latest ones were unacceptable, probably due to irreproducible derivatization. For all other analytes, the method was linear from 5 to 500 ng/mL.

A GC–MS method, based on derivatization with 2,2,2-trichloroethyl chloroformate, was investigated by Frison et al. [109] for determination of some 2,5-dimethoxyamphetamines (2,5-DMA, DOM and DOB) in plasma, urine and hair samples. Sample preparation involved alkaline extraction from biological samples using Extrelut columns. Hair samples were previously decontaminated and incubated overnight at 45 °C with acidic methanol. The subsequent derivatization with 2,2,2-trichloroethyl chloroformate gave distinctive MS spectra with characteristic isotopic clusters that allow unambiguous analyte identification. This results in a potentially better chromatographic selectivity when analysing complex biological matrices. Quantitative studies using select ion monitoring (SIM) conditions gave a linear response over ranges of 10–2000 ng/mL (plasma and urine) and 0.20–20 ng/mg (hair). This is a preliminary method that needs of a full validation prior to be applied in clinical and forensic analysis.

Kanai et al. [110] compared a GC–MS method with and without trifluoroacetyl derivatization for the simultaneous analysis of six phenethylamine designer drugs (2C-H, 2,5-DMA, 2C-B, DOB, 2C-I, and DOI). The purposed method was designed for identification and quantification in seized tablets; therefore, highly sensitive detection techniques such as SIM were not employed, and the calibration curves were set at high levels. Anyway, the research of Kanai et al. was reported in this review, because it compared a GC–MS method with and without derivatization. The authors concluded that the GC–MS analysis of underivatized compounds was not suitable for the simultaneous determination of 2,5-dimethoxyphenethylamines.

### Capillary electrophoresis

Capillary electrophoresis (CE) analysis has been first used by Nieddu et al. [111, 112] for simultaneous determination of ten 2,5-dimethoxy substituted amphetamines (Table 1) using two different detection methods, diode array detector (DAD) and mass spectrometry (MS), in blood and urine analysis, respectively. The clean-up procedure from blood was carried out using a liquid/liquid extraction (LLE) with acetonitrile, previously described for other amphetamines [113], while for urine samples, a solid-phase extraction (SPE) with Bond Elut C18 cartridges was specifically validated [112]. The limits of quantification (LOQs) by CE–DAD were sufficient to detect the presence of these analytes in blood after acute exposure. The method was in vivo applied in rats after a single intraperitoneal administration (1 mg/kg), providing realistic drug concentrations in case of fatal intoxication [76]. With regard to DAD detection, the use of MS detector allowed to obtain much more lower LOQs, ranging from 1 to 14 ng/mL [112]. In addition, CE–MS analysis provided specific mass spectra that permit the unambiguous confirmation of these drugs, and could be useful not only in urine, but also in other biological matrices as well in confiscated tablets. Several of the investigated amphetamines are scheduled in the US and in European Union (EU) [17, 18, 114–116].

CE–MS has been also applied by the same authors to identify four compounds of 2C-T series (2C-T, 2C-T-2, 2CT-5 and 2C-T-7) and related thio-amphetamines (ALEPH, ALEPH-2, ALEPH-5 and ALEPH-7) in human plasma (Table 1) [117, 118]. The 2C-T-2 and 2C-T-7 are included in the list of narcotic substances in several countries [17, 18, 114]. The extraction of 2C-T derivatives from plasma was carried out using an extractive procedure already described for other amphetamines [111, 113]; for ALEPH compounds, a SPE clean-up previously validated in urine samples was used [112]. CE separations were performed using 10 mM phosphate buffer pH 2.5. For all analysed substances, MS detection permitted to obtain LOQs in order of ng/mL (Table 1), enough for confirmatory testing of plasma levels of drug consumers.

Similar CE–MS methods were validated for another group of 2C-T derivatives (2C-T-4, 2C-T-8, 2CT-13 and 2C-T-17) and related ALEPHs in human urine [119, 120]. CE conditions were optimized modifying the background electrolyte and electro-osmotic flow. A buffer of 50 mM ammonium acetate (pH 4.5) and separation voltage of 25 kV were used. The validation of method involved measurements of the following parameters: selectivity, linearity, limits of detection and quantification, recovery, accuracy, precision, matrix effect and sample stability. All parameters were within the required limits [121, 122]. LOQ values were comparable with those observed for similar amphetamines by CE–MS, and suitable for urine confirmatory tests (Table 1).

CE–MS has been also applied by the same authors to identify three 4-alkyl substituted 2,5-dimethoxy-amphetamines (DOM, DOET and DOPR) in urine samples (Table 1) [123]. Electrophoretic separation was performed using a pH 4.5 buffer. A simple SPE clean-up allowed to obtain electropherograms free from interfering peaks. The method was validated according to international guidelines [121, 124]. The calibration curves showed linearity in the range of 10–1000 ng/mL for all analysed amphetamines.

CE–MS was demonstrated to be an interesting alternative to GC–MS and an elective technique for amphetamine derivatives analyses, because it requires less sample manipulation and shorter analysis times.

### Liquid chromatography

Liquid chromatography–tandem mass spectrometry (LC–MS/MS) has proved to be a better alternative than CE–MS and GC–MS, for the analysis of phenethylamines in biological matrices.

In 2009, Nieddu et al. [125] first reported a rapid LC–MS/MS method for the simultaneous determination of eight thio-amphetamines and phenethylamines (Table 1) in human urine. The same compounds had been previously detected in plasma by CE–MS analysis using two separate chromatographic runs [117, 118]. Unlike the latter, LC–MS/MS permitted to separate more easily congeners with the same molecular mass, improving the selectivity of the method and permitting to separate simultaneously all eight congeners. The SPE procedure used for clean-up and pre-concentration of the samples had been already validated [112, 118, 123, 126]. The method was proven to be comparable in accuracy and precision with those CE–MS designed for the same compounds. The limits of sensitivity are better than those reported with CE–MS analysis (Table 1) and more suitable for monitoring of these analytes in urine samples.

Another study on the simultaneous LC–MS/MS determination of 2,5-dimethoxy-derivatives in human urine is that of Poklis et al. [28] in 2014, concerning 25-NBOMe derivatives (Table 1). The NBOMe designer drugs are rapidly extracted from urine by SPE with FASt^™^ columns. The method has been fully validated for linearity, LOQ, limit of detection (LOD), accuracy/bias, precision, dilution integrity, carryover, selectivity, ion suppression and stability. The same authors previously published LC–MS/MS methods addressed to the detection of only one or two NBOMe derivatives in some cases of severe or fatal intoxication [9, 127–129]. These methods included only limited validation data as parts of case reports.

Regarding blood samples analysis, Adamowicz and Tokarczyk (2015) [130] validated a rapid screening for 143 psychoactive substances, including 13 compounds belonging to the 2,5-dimethoxy-phenylethylamines group (Table 1). A simple deproteinization with acetonitrile was need for blood samples clean-up. However, validation method was performed only for 32 out of 143 tested compounds. Calibration curves were linear in the range of 1–100 ng/mL and the procedure was successfully applied to routine analysis of forensic cases.

In 2015, Montenarh et al. [131] developed a LC–MS/MS screening method for the detection of 130 different analytes, among them 2C-P, 2C-B, 2C-D, 2C-E, 2C-I and 2C-T, in different biological specimens (blood, plasma, serum, postmortem blood, liver tissue, gastric contents, hair, and urine). Samples were extracted with diethyl ether/ethyl acetate mixture (1:1, v/v) at different pH values, depending on analysed matrix. One single work-up approach, adopted for all biological samples, did not provide a full validation for all 130 analytes. Regarding substance topic of this review, recovery and precision data were given only for the 2C-P and with precision values falling out of the acceptable criteria for the high control samples. Whereas, the LODs were provided for all investigated substances, and ranged from 10 to 50 ng/mL (Table 1). The multi-drugs procedure was applied on more than 900 authentic samples, but none of 2C compounds were found.

In 2017, Abbara et al. [12] reported a validated LC–MS/MS method for the analysis of 2,5-DMA, DOI, DOC, DOB, DOM, and DOET, in plasma and urine samples of five patients with non-fatal intoxication by amphetamine derivatives. The analysis confirmed the consumption of DOC by all patients, with plasma concentrations around LOQ (10 ng/mL), and urine concentrations ranging from 300 to 1300 ng/mL.

Six studies were found for multi-drugs detection of 2,5-dimethoxy-amphetamine designer drugs in hair [88, 132–136]. An LC–MS/MS method for the simultaneous analysis of opiates, cocaine and amphetamines in hair samples was presented by Imbert et al. in 2014 [132]. This is the first method that allowed the detection of two amphetamine designer drugs of DOx series (DOB and DOM) in hair. Hair samples, previously decontaminated by washing with water and dichloromethane, were incubated for 18 h at 45 °C with phosphate buffer (pH 5.0), and then purified by SPE clean-up. The validation procedure included linearity, intraday and interday accuracy and precision. A value of 0.05 ng/mg was achieved for the LOQ, in accordance with the values recommended by the Society of Hair Testing (SoHT) on hair testing in forensic cases, which required an LOQ of almost 0.2 ng/mg for amphetamines [137]. This method was validated with four external quality controls by the German Society of Toxicological and Forensic Chemistry (GTFCh) and three by the SoHT. Finally, the validated method was applied to authentic forensic cases.

In 2015, Nieddu et al. [88] reported a simple procedure for the simultaneous determination in hair of 11 illicit phenethylamines (Table 1) by LC–MS/MS analysis. The method was validated according to the SoHT guidelines for drug testing in hair [42]. Extraction from hair was performed after incubation in methanolic HCl at 45 °C for 24 h. The LOQs, ranging from 0.09 to 0.20 ng/mg, are suitable to detect the presence of these analytes in toxicological and forensic samples, according to hair cutoff value established for similar amphetamines [137]. The method was applied in vivo on rats in order to investigate the effect of the pigmentation on drugs distribution between pigmented and non-pigmented hair. The investigated phenethylamines were found only in pigmented hair, confirming that basic substances are incorporated more easily in pigmented hair than in non-pigmented ones, as already reported [91–93]. In light of these results, when determining basic drugs, it should be recommended to perform the analysis on pigmented hair or, in absence of them, it would be advisable to establish different cutoff values on the basis of hair pigmentation.

In 2016, Salomone et al. [133] developed an LC–MS/MS assay for the determination of 31 new designer drugs in hair matrices. Two substances of 2C series (2C-P, 2C-B) and four of NBOMe group (25I-NBOMe, 25C-NBOMe, 25H-NBOMe, 25B-NBOMe) were tested. A simple pre-treatment in methanol at 55 °C for 15 h had been employed. Selectivity, specificity, linearity range, LOD and LOQ, intra-assay and inter-assay precision and accuracy, carryover effect, recovery, and matrix effect were investigated for full validation of the method. LOQ values ranged between 2 and 12 pg/mg for all investigated substances**.** The application to real cases did not detected substances of 2C series in any of the considered samples. The authors attributed the negative results probably to the great pharmacological activity of these designer drugs that need very low doses, reducing the detectable concentrations in hair, especially in cases of sporadic intake**.** From here, the need for further improvement of the sensitivity of the method to disclose possible presence at traces in hair.

Boumba et al. (2017) [134] described a rapid LC–MS/MS method for the screening of 132 NPS, including eight amphetamine-type stimulants (Table 1). The extraction procedure from hair utilized a single incubation step with HCl in methanol (at 40 °C for 3 h) for all different classes of substances, including unstable compounds (cathinones) and hydrophobic compounds (synthetic cannabinoids). The method was validated according to Scientific Working Group for Forensic Toxicology (SWGTOX) [138]. Concerning analytes of interest of this review, validation criteria were satisfactory. Over a total of 23 investigated real cases, 2,5-DMA and 25C-NBOMe were found in two different hair samples, respectively.

A multi-class analysis of NPS in hair samples by pressurized liquid extraction (PLE) was developed by Montesano et al. in 2017 [135]**.** The present method was primarily addressed to analysis of cathinones and synthetic cannabinoids, but a phenethylamine (2C-T4) was included in order to demonstrate that PLE coupled to SPE clean-up is suitable for a multi-class analysis. The method was fully validated according to accepted guidelines [42, 138]. The use of PLE allowed a significant reduction of the long incubation times of classical hair digestion. The entire procedure required approximately 45 min for decontamination, incubation, clean-up, and LC–MS/MS analysis. In addition, PLE seemed to be more appropriate than hair digestion for multi-class analysis considering that several compounds (e.g., cathinones) are not stable under the strong alkaline or acidic conditions. More recently, the same authors proposed a further improvement of the extraction method, using a combination of PLE with dispersive liquid–liquid micro extraction (dLLME), for multi-class analysis of drugs of abuse in hair [136]. Furthermore, the number of analysed designer drugs was implemented, including also five compounds of 2C series (Table 1). The clean-up through dLLME, compared to SPE, reduced amount of solvent, cost and analytical times. PLE-dLLME procedure showed to be suitable for multi-class extraction from hair, resulting in reproducible results with significant reduction of analysis times. The method, fully validated following SWGTOX guidelines [138], was successfully applied in forensic applications but no phenethylamine derivatives were found.

Concerning alternative matrices, two studies of our research team about the detection of a group of 2,5-dimethoxyamphetamine designer drugs (Table 1) in amniotic fluid [139] and oral fluid [140] were reported. The authors used a LC–MS/MS method previously validated in hair for the same group of compounds. Both analytical procedures were validated in terms of selectivity, linearity, LOD and LOQ, precision, accuracy, matrix effect and analyte stability, according to accepted guidelines [121, 122, 124, 141]. Regarding amniotic fluid, a simple SPE with hydrophilic-lipophilic balance (HLB) cartridges gave good recoveries and low matrix effects [139]. For oral fluid samples, a new extractive approach has been used applying supramolecular solvents (SUPRAS) [140]. SUPRAS are tailored solvents that can be totally modulated by selecting synthesis conditions. They are nanostructured systems generated by a spontaneous mechanism of self-assembly and coacervation of a colloidal solution of amphiphiles. In oral fluid, the synthesis of SUPRAS is directly conduced in sample because of its high content of water (99.5%) [142]. In this study, SUPRAS was generated from mixture of hexanol/tetrahydrofuran (THF)/oral fluid, achieved by adding colloidal solution of hexanol in THF to oral fluid. The generated SUPRAS showed an hexagonal nanostructure with different polarity regions that allowed analytes interacted in the mixed-mode, with the alcohol groups of the hexanol that surround water cavities, and with C-chains facing towards THF. The typical matrix interferences, as proteins and carbohydrates, were removed during clean-up by mechanisms of precipitation, flocculation or size exclusion [142]. Compared to previous extraction methods from oral fluid, SUPRAS approach was proved to be more efficient in removing matrix effect, with further improvement of LOQ values (Table 1).

In the last years, the research of new analytical methods is focusing mainly towards newly emerging designer drugs, as NBOMe and NBOH compounds. The LC–MS/MS is the most used method to identify these classes of substances [143–152]. In particular, considering the thermal lability of NBOHs, the LC–MS/MS allows to prevent their misidentification with the corresponding 2C compounds, as happened when GC–MS was used as analytical detection.

In 2018, the forensic toxicology laboratory of Nassau Medical Examiner developed a sensitive LC–MS/MS method to identify 50 illicit substances in postmortem blood and urine samples [144]. Between the investigated substances, also some 2,5-dimethoxy-amphetamines and phenethylamines (25B-NBOMe, 25C-NBOMe, 25I-NBOMe, 25I-NBOH, 2C-C, 2C-I, 2C-E, DOI, and DOB) were included. Sample preparation was based on a simple LLE and the method was validated for sample stability, selectivity/specificity, matrix effect, carryover, and LOD (Table 1) according to SWGTOX guidelines [138].

Another LC–MS/MS method was developed and validated for qualitative analysis of 51 NPS in whole blood by Franck et al. [145]. Several NBOH and NBOMe derivatives were included in the method (Table 1). Blood extraction was carried out by a LLE with dichloromethane/butyl chloride (1:4, v/v). The assessed validation parameters were specificity, LOD, precision, stability and matrix effect.

In 2019, Ng et al. [146] developed and validated a method for the simultaneous analysis of synthetic hallucinogens (25C-NBOMe, 25B-NBOMe, 25I-NBOMe, 25H-NBOMe, 25C-NBOH, 25B-NBOH, 25I-NBOH and 25H-NBOH) in urine. The method was validated for extraction recovery, matrix effect, accuracy and precision, LOD, carryover and stability. Urine samples were extracted using supported-liquid extraction (SLE) on a Biotage Isolute cartridge, obtaining recoveries over than 80% for the NBOMe and NBOH analogues. LOD values were of 0.5 ng/mL for all 2,5-dimethoxy-derivatives except for 25C-NBOMe, 25H-NBOMe and 25H-NBOH, which showed higher LODs (1 ng/mL), probably due to matrix interference. The method was also successfully applied to authentic urine samples from suspected drug abusers.

In a study of Cheng et al. of 2020, the prevalence of drugs of abuse detected in Hong Kong from 2016 to 2018 has been investigated on seizures and urine samples. One of the limitations of this study is that analysis of NPS was not included in routine urine testing. Between NPS identified in seizures, there were also 2C-B, 25I-NBOMe, 25I-NBOH and 25C-NBOH [147].

A similar study was conducted in Brazil by da Cunha et al. in 2021 [148]. The prevalence of NPS has been evaluated through the analysis of oral fluid samples collected at electronic music festivals and parties. Toxicological analysis revealed the presence of 25I-NBOH, 25C-NBOH, 25B-NBOH, and 25I-NBOMe in several oral fluid samples. Detailed information regarding chromatographic conditions and validation data had been previously published by the same authors [149]. Over 100 NPS, including 22 phenethylamines (Table 1), were analysed by a LC–MS/MS method validated following the SWGTOX guidelines [138]. The Quantisal^™^ device was successfully used to collect oral fluid samples. Sample extraction has been carried out by a simple LLE procedure, less expensive of SPE clean-up. Extraction recoveries were over than 70% for all phenethylamines, except for 2C-D (66.2%), 2C-T (47.4%) and 2C-T-2 (63.2%). LOD values ranged from 0.05 to 1 ng/mL (Table 1).

In 2021, Breusova et al. [150] validated an LC–MS/MS method for the quantification of the 4-cyano-2,5-dimethoxy-*N*-(2-hydroxybenzyl)phenethylamine (25CN-NBOH) and its metabolite 2C-CN in rat plasma and brain. For samples clean-up, a new hybrid technique, which simultaneously removes proteins and phospholipids (PP), was tested. Particularly brain tissue is rich in PP, which can negatively affect LC–MS analysis. The “Phree PP Removal” from Phenomenex^®^ was proven to be an efficient extractive method, less expensive and time-consumption than other purification methods [150]. It provided good recoveries between 75.2 and 94.2% for both studied compounds. Accuracy, precision, recovery, matrix effect, selectivity, LOD and LOQ, linearity, and stability were assessed for method validation. The LOQs for 25CN-NBOH and 2C-CN were 1 ng/mL and 5 ng/100 mg for plasma and brain, respectively (Table1).

The LC–MS/MS method proposed by Fan et al. [151] permits the simultaneous screening of 74 phenethylamines in urine samples, including several 2,5-dimethoxy-amphetamines and -phenethylamines (Table 1). Urine samples were analysed using a dilute procedure without any purification. The method was validated in terms of carryover, selectivity, linearity, sensitivity, matrix effect, precision, and accuracy [138]. Regarding carryover, the authors took attention about some 25-series phenethylamines (25G-NBOMe, 25C-NBOMe, 25P-NBOMe, 25N-NBOMe, 25T7-NBOMe, 25B-NBOMe and 25I-NBOMe) that ranged in 25.9–71.3%, indicating the residue appearing in the subsequent blank. To avoid false-positive results from the residue of the preceding sample, it should be sufficiently eluted until no residue was observed in the blank. The method was proven to be selective for all analytes in a linearity range of 1.0–50.0 ng/mL. The LOQs for all analytes were 1.0 ng/mL. The validated method was applied to authentic urine samples, but no 2,5-dimethoxy derivatives have been found.

Also in 2021, DiRago et al. [152] described a rapid technique for detection and semi-quantification of 327 drugs in blood, using a one-step liquid extraction and automated data processing to yield rapid analysis times. The LC–MS/MS, fully validated in accordance with internationally accepted guidelines [122, 138], permits a rapid analysis of comprehensive range of drugs of abuse, including various 2,5-dimethoxy-phenetylamines (2C-B, 2C-I, 2C-N, 25B-NBOMe, 25C-NBOMe, and 25I-NBOMe). The technique was proven to be sufficiently rapid and reliable for forensic casework with good LOQ values for the analyte topic of this review (Table 1). The application to numerous forensic cases allowed identifying a case of intoxication by 25C-NBOMe, in which a blood concentration of 0.002 mg/L was found.

In 2022, Ferrari et al. [153] published a LC–MS/MS method for analysis of 79 NPS in postmortem blood and urine samples. Among them twelve 2,5-dimethoxy-derivatives were extracted from biological matrices by a QuEChERS (Quick, Easy, Cheap, Effective, Rugged, and Safe) protocol, obtaining good recoveries for all the analytes of interest (mean recovery > 85%). The method was validated for selectivity, matrix effect, linearity, recovery, accuracy, precision, carryover and sample stability [154]. The group of NBOHs and NBOMes showed LOQ values (0.4 ng/mL) lower than corresponding 2C compounds (Table 1). Given the high pharmacological activity of these compounds, it is a great advantage to have more sensitive analytical methods.

In a very recent study, Hwang et al. [155] have reported a new screening of 40 NPS in human plasma using a magnetic solid-phase extraction followed by liquid chromatography quadrupole time-of-flight mass spectrometry (LC–QTOF-MS). The extractive method is based on the use of a magnetic sorbent dispersed in the sample solution that can be separated by an external magnetic field due to the presence of magnetic nanoparticles. This technique proved to be an efficient method to extract phenethylamines from human plasma. The validation data for phenethylamines showed acceptable results for recoveries (76.0–102%), matrix effects (− 14.9 to 6.1%), and precision (2.1–16.8%). The LODs ranged from 2 to 27 ng/mL (Table 1).

## Conclusions

The presence of updated analytical procedures for the identification of drugs of abuse in biological matrices is essential for toxicological analysis. In particular, considering the lack of available screening immunoassays in detecting most of new amphetamine derivatives, it is of crucial importance to develop new analytical methods able to identify these substances in the biological specimens. The non-detection of legally controlled substances can lead to erroneous estimation of their global use. The validation of new methods for the determination of phenethylamine analogues has exponentially increased over these years, along with the rapid growth in the number of clinical and forensic positive cases.

The present review provides an updated overview of the analytical procedures designed to confirm the presence of 2,5-dimethoxy-amphetamines and -phenethylamines in biological samples. The choice of the biological matrix depends on the purpose of the survey and on the basis of information assembled. Nevertheless, the knowledge of drugs consumption history can help to clarify the interpretation of toxicological findings.

Blood or plasma analysis is preferred to evaluate a short-term intake, while urine is the matrix of choice for preliminary screening of a wide range of substances and their metabolites, even several days after intake. The use of alternative biological matrices, as oral fluid or hair, permits to expand the knowledge in a context of acute or chronic intoxications, respectively. Hair analysis, particularly increased in the last decades, presents two major criticisms: the limited amount of samples available and the low concentrations of analytes. The new type of LC–MS/MS techniques permits to obtain good results in such challenging samples.

From the analyses of the processed data, it emerges that LC–MS/MS is a better tool than CE–MS and GC–MS, as it permits to separate more easily congeners with the same molecular mass, improving the selectivity of the method. In addition, a great advantage, when compared with GC analysis, is that it prevents the thermal decomposition of the newly emerging designer drugs of NBOH series.

Regarding 2C and DOx series compounds, many LC–MS/MS methods for analysis in biological matrices, both traditional and alternative, are available, with LOQs ranging from 1.0 to 20.0 ng/mL for biological fluids and, from 0.002 to 0.2 ng/mg for hair matrix. In general, the NBOMe and NBOH compounds show lower LOQ values. This is an important goal considering that they are highly active drugs and are taken at very low concentrations.

The topic is constantly evolving due to the rapid circulation of NPS. It would be undoubtedly useful to have a multiresidue method, validated on multiple matrices at the same time, in order to obtain a forensic analysis as more reliable as possible.
